# Altitude Acclimatization Alleviates the Hypoxia-Induced Suppression of Exogenous Glucose Oxidation During Steady-State Aerobic Exercise

**DOI:** 10.3389/fphys.2018.00830

**Published:** 2018-07-09

**Authors:** Andrew J. Young, Claire E. Berryman, Robert W. Kenefick, Allyson N. Derosier, Lee M. Margolis, Marques A. Wilson, Christopher T. Carrigan, Nancy E. Murphy, John W. Carbone, Jennifer C. Rood, Stefan M. Pasiakos

**Affiliations:** ^1^Military Nutrition Division, United States Army Research Institute of Environmental Medicine, Natick, MA, United States; ^2^Oak Ridge Institute of Science and Education, Oak Ridge, TN, United States; ^3^Thermal Mountain and Medicine Division, United States Army Research Institute of Environmental Medicine, Natick, MA, United States; ^4^School of Health Sciences, Eastern Michigan University, Ypsilanti, MI, United States; ^5^Pennington Biomedical Research Center, Baton Rouge, LA, United States

**Keywords:** high-altitude, substrate metabolism, carbohydrate supplementation, aerobic exercise, insulin

## Abstract

This study investigated how high-altitude (HA, 4300 m) acclimatization affected exogenous glucose oxidation during aerobic exercise. Sea-level (SL) residents (*n* = 14 men) performed 80-min, metabolically matched exercise ($\dot{V}$O_2_ ∼ 1.7 L/min) at SL and at HA < 5 h after arrival (acute HA, AHA) and following 22-d of HA acclimatization (chronic HA, CHA). During HA acclimatization, participants sustained a controlled negative energy balance (-40%) to simulate the “real world” conditions that lowlanders typically experience during HA sojourns. During exercise, participants consumed carbohydrate (CHO, *n* = 8, 65.25 g fructose + 79.75 g glucose, 1.8 g carbohydrate/min) or placebo (PLA, *n* = 6). Total carbohydrate oxidation was determined by indirect calorimetry and exogenous glucose oxidation by tracer technique with ^13^C. Participants lost (*P* ≤ 0.05, mean ± SD) 7.9 ± 1.9 kg body mass during the HA acclimatization and energy deficit period. In CHO, total exogenous glucose oxidized during the final 40 min of exercise was lower (*P* < 0.01) at AHA (7.4 ± 3.7 g) than SL (15.3 ± 2.2 g) and CHA (12.4 ± 2.3 g), but there were no differences between SL and CHA. Blood glucose and insulin increased (*P* ≤ 0.05) during the first 20 min of exercise in CHO, but not PLA. In CHO, glucose declined to pre-exercise concentrations as exercise continued at SL, but remained elevated (*P* ≤ 0.05) throughout exercise at AHA and CHA. Insulin increased during exercise in CHO, but the increase was greater (*P* ≤ 0.05) at AHA than at SL and CHA, which did not differ. Thus, while acute hypoxia suppressed exogenous glucose oxidation during steady-state aerobic exercise, that hypoxic suppression is alleviated following altitude acclimatization and concomitant negative energy balance.

## Introduction

Ingesting carbohydrate during endurance exercise at sea level (SL) increases exogenous carbohydrate oxidation and limits endogenous carbohydrate utilization (Jeukendrup et al., 1997; Jeukendrup, 2004, 2008). Only two studies have investigated how hypoxia affects oxidation of exogenous carbohydrate ingested during exercise, and the observations reported from those studies differ (Peronnet et al., 2006; O’Hara et al., 2017). When carbohydrate was ingested during exercise at 77% $\dot{V}$O_2max_ in normoxia and hypoxia equivalent to 4,300 m, exogenous carbohydrate oxidation was the same (Peronnet et al., 2006). In contrast, when carbohydrate was ingested during lower intensity exercise (55% W_max_, ∼55–60% $\dot{V}$O_2max_), exogenous carbohydrate oxidation was lower at high-altitude (HA; 3,375 m) than normoxia (O’Hara et al., 2017). The reasons for the discrepant effects of hypoxia observed in these studies on exogenous carbohydrate oxidation during exercise are not clear, but might reflect differences in the exercise intensities employed.

Hypoxia reduces $\dot{V}$O_2max_ (Young et al., 1985; Young and Reeves, 1998), so in the experiments described above (Peronnet et al., 2006; O’Hara et al., 2017), exercise was performed at lower absolute intensity and metabolic rate in hypoxia so as to maintain relative exercise intensity (i.e., % $\dot{V}$O_2max_) the same as it was in normoxia. However, from a scientific (Brooks et al., 1991; Roberts et al., 1996) and practical perspective (the metabolic rate during physical activities is the same at SL and HA, notwithstanding differences in relative intensity, i.e., % $\dot{V}$O_2max_), we believe that differences in substrate metabolism during exercise at HA and SL should be investigated at the same absolute intensity and metabolic rate in both conditions. In one of the aforementioned studies, Peronnet et al. (2006) also compared exogenous carbohydrate oxidation during exercise performed at the same absolute intensity and metabolic rate (i.e., $\dot{V}$O_2_ = ∼2.2 L/min, 11.6 kcal/min) in normoxia (54% $\dot{V}$O_2max_) and hypoxia equivalent to 4,300 m (78% $\dot{V}$O_2max_), and reported that exogenous carbohydrate oxidation was again the same in hypoxia as normoxia, despite increases in both total and endogenous carbohydrate oxidation in hypoxia. Maximal exogenous carbohydrate oxidation rates are achieved between 51 and 64% of $\dot{V}$O_2max_ (Pirnay et al., 1982). Therefore, in that experiment (Peronnet et al., 2006), it is possible that exogenous carbohydrate oxidation was already maximal during the normoxic exercise trials and could not increase any further during the hypoxic exercise. Whether oxidation rate of exogenous carbohydrate ingested would differ during exercise performed at the same absolute intensity in normoxic and hypoxic conditions at exercise intensities eliciting less than maximal exogenous carbohydrate oxidation rates at HA has not been determined. In addition, previous investigations of the effect of hypoxia on exogenous carbohydrate oxidation during exercise have studied unacclimatized lowlanders (Peronnet et al., 2006; O’Hara et al., 2017), and how altitude acclimatization affects exogenous carbohydrate oxidation during exercise is not known.

Sojourners at HA experience both anorexia and increased total daily energy expenditure (TDEE) (Westerterp et al., 1994; Butterfield, 1999). The resultant negative energy balance and associated loss of body mass during HA sojourns lasting longer than several days (Butterfield et al., 1992), could also influence changes in substrate metabolism developing during HA acclimatization. While imposing strict dietary controls can attenuate daily energy deficits and partially mitigate weight loss (Butterfield et al., 1992), most individuals fail to consume sufficient food to maintain energy balance during HA sojourns and, thus, experience weight loss (Rose et al., 1988; Hoyt et al., 1994). As such, the metabolic effects of putative nutrition interventions for HA sojourns should be evaluated in response to the “real world” conditions that lowlanders typically experience during prolonged HA sojourns (i.e., negative energy balance).

Thus, the objective this study was to examine how the oxidation of exogenous carbohydrate by lowlanders performing metabolically matched, steady-state aerobic exercise was affected by acute HA (AHA) exposure (<5 h) and 22 days of HA acclimatization and negative energy balance. In our experiments, exercise was performed at an intensity selected to elicit less than maximal rates of exogenous carbohydrate oxidation (Pirnay et al., 1982). We hypothesized that exogenous carbohydrate oxidation in lowlanders consuming carbohydrate during exercise in hypoxic conditions would be the same or lower during acute hypoxic exposure compared to exercise at SL, and that exogenous carbohydrate oxidation would increase with HA acclimatization.

## Materials and Methods

### Participants

Participants in this study were part of a larger randomized, controlled study that assessed the effects of standard (1.0 g/kg/d) versus higher (2.0 g/kg/d) protein diets on body composition in response to 21 days of negative energy balance at HA (Berryman et al., 2018). In brief, 17 recreationally active (programmed physical activity 2–4 d/week) men, aged 18–42 years who were not acclimatized to HA (i.e., born at altitudes < 2,100 m, currently residing at altitudes < 1,200 m, and who had not traveled to altitudes > 1,200 m for 5 days or more within 2 months before beginning the study) completed that parent study (Berryman et al., 2018). To address whether AHA exposure, and acclimatization to HA, affected exogenous glucose oxidation during aerobic steady-state exercise, participants assigned to the standard (*n* = 8) and higher (*n* = 9) protein diets were further randomized to groups provided equal volumes of flavor-matched carbohydrate (CHO; *n* = 9, 4 standard and 5 higher protein) and placebo (PLA; *n* = 8, 4 standard and 4 higher protein) drinks during exercise. During data analysis, it was discovered that ambient conditions were inaccurately entered for one of the metabolic carts used for testing three participants during one of the substrate oxidation experiments, so all data for those participants were excluded from the analysis. Data from the remaining 14 participants (CHO; *n* = 8, 3 standard and 5 higher protein; and PLA; *n* = 6, 4 standard and 2 higher protein) are presented in this report. All volunteers provided written informed consent prior to participation. This study was approved by the Institutional Review Board at the United States Army Research Institute of Environmental Medicine (USARIEM, Natick, MA, United States) and registered at www.clinicaltrials.gov as NCT02731066. Investigators adhered to the policies for protection of human subjects as prescribed in the US Department of Defense Instruction 3216.02, and the research was conducted in adherence with the provisions of 32 CFR Part 219.

### Experimental Design and Diet

**FIGURE 1:**
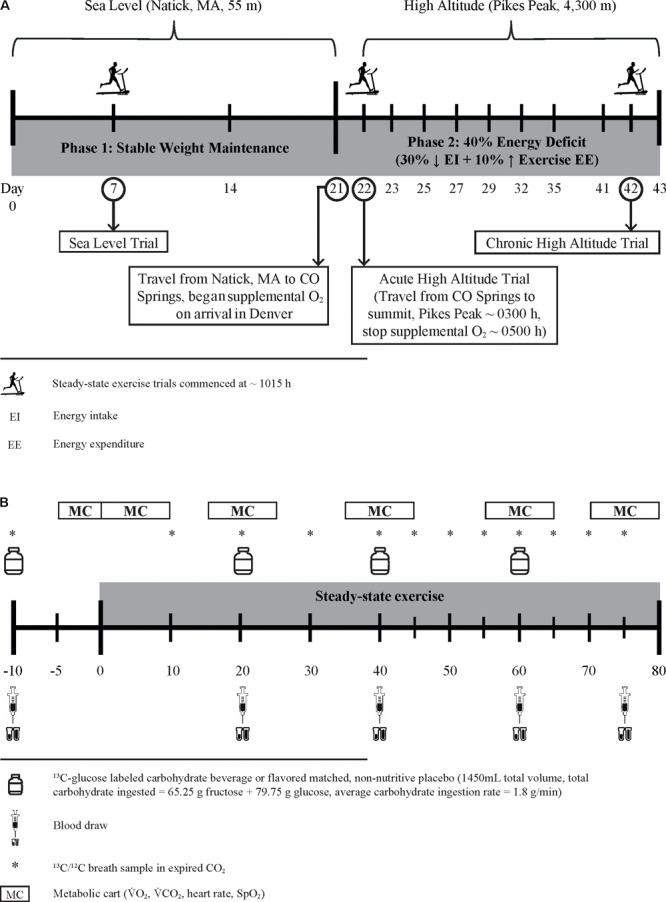
Experimental design schematic **(A)** and protocol for assessment of substrate oxidation and blood metabolite changes during steady-state exercise **(B)**.

This 43 consecutive day controlled study included three distinct phases; 21 days of energy balance at sea level (SL, 1–21), 1 day of AHA exposure (day 1), and 21 days of energy deficit at HA (days 2–22) (**Figure 1A**). Substrate oxidation responses to CHO and PLA ingestion during steady-state exercise were assessed beginning at 10:15–11:15 h at SL day 7, AHA, and on day 22 of chronic HA exposure (CHA). During SL, Registered Dietitians instructed (i.e., what foods to eat, how much, what foods to avoid, etc.) participants to follow a weight-maintaining diet providing 1.0 g protein/kg/d, 45–65% total energy from carbohydrate, and 20–35% total energy from fat. The protein intake level was chosen based on mean *ad libitum* protein intakes in active duty military personnel (Margolis et al., 2012). TDEE was estimated from the sum of (a) measured resting metabolic rate (multiplied by 1.3 to account for activities of daily living and diet-induced thermogenesis) and (b) exercise-induced energy expenditure (EIEE) estimated from corresponding metabolic equivalents of activities reported in the 3 days physical activity log (Ainsworth et al., 2011; Berryman et al., 2018). Those TDEE estimates were then averaged with estimated TDEE requirements calculated using the Harris–Benedict equation (Roza and Shizgal, 1984). Participants were asked to maintain their self-reported exercise routine for the duration of SL testing, although participants were prohibited from performing any exercise within 24 h of the substrate oxidation experiments. Compliance with diet and activity instructions were verified using 24 h diet records, activity recalls, and by measuring body mass daily, upon waking (overnight fast, ≥8 h), and after voiding, to the nearest 0.1 kg using a calibrated digital scale (Befour model PS6600; Befour Inc., Saukville, WI, United States).

On day 21, participants were flown to Denver, CO (1,630 m) and transported by research staff to Colorado Springs, CO (1,840 m), where they stayed overnight, under supervision, in a local hotel before being transported (∼03:00 h) to the USARIEM Maher Memorial Altitude Laboratory on the summit of Pikes Peak, CO (4,300 m, day 22). Participants breathed supplemental oxygen (portable O_2_ concentrators, FreeStyle 5, AirSep, Buffalo, NY, United States or EverGo, Respironics, Murrysville, PA, United States) after landing in Denver until they arrived at the summit about 13-h later, so as to maintain normoxic oxygen saturation (mean ± SD for peripheral oxygen saturation [finger pulse oximetry, Nonin, Model 9560, Plymouth, MN, United States] measured hourly from arrival until 22:00 h and every 30 min beginning at 03:00 h was 97.0 ± 0.9%, range 95–99). This allowed the initiation of hypoxic exposure before experimental testing began to be standardized among all of the participants (∼315 min). Participants did not breathe supplemental oxygen until they arrived in Denver, and thus experienced a mild, 4-h hypoxic exposure equivalent to ∼1,900 m (Hampson et al., 2013) during the 4-h flight from Boston. Starting the morning following the AHA experiment, participants were randomized and consumed either the standard or higher protein [amount consistent with recommendations for strenuous military operations, (Pasiakos et al., 2013a)] diet for the next 21 days (HA days 2–22). The carbohydrate content of the diets was fixed at 45% of total energy consumed so as to avoid confounding effects of variations in daily carbohydrate intake on substrate oxidation during exercise during the CHA trial. The remainder of the energy consumed was derived from fat. For HA, Registered Dietitians created diets based on individualized energy requirements to maintain body mass at SL, consisting primarily of components of the military combat ration, Meal, Ready-to-Eat (MRE; menu 34; AmeriQual, Evansville, IN, United States). The remainder of the energy was derived from perishable foods and whey protein drinks (Isopure^®^ Zero Carb), as previously described (Berryman et al., 2018). All foods and caloric drinks were weighed to the nearest 0.1 g, and preparation and consumption of all meals and snacks were supervised by trained research staff. Non-caloric, non-caffeinated drinks were allowed *ad libitum*.

Daily energy intake during the HA phase was reduced by 30% relative to estimated TDEE required to maintain stable body mass at SL, and EIEE was increased 10% above SL TDEE [i.e., HA EIEE = SL EIEE + (0.1 × SL TDEE)] (Berryman et al., 2018). This was done to elicit an energy deficit consistent with our previous work (Pasiakos et al., 2013b, 2014) and equivalent to the deficit commonly experienced by Soldiers subsisting solely on military rations during extended-duration combat operations and training exercises (Tharion et al., 2005; Margolis et al., 2014). Exercise sessions included low to moderate intensity treadmill walking, running, and outdoor hiking, all supervised and documented by research personnel to ensure safety and compliance with the exercise prescription. The amount of physical activity necessary to elicit the desired daily EIEE during altitude acclimatization was individually prescribed, and determined using the Pandolf Equation for level and uphill walking (Pandolf et al., 1977), adjusted as necessary for downhill walking (Santee et al., 2003). Changes in body mass and composition during HA acclimatization phase are presented and discussed in detail separately (Berryman et al., 2018). In this report, only body mass changes will be presented so as to provide perspective for the controlled energy deficit.

### Peak Oxygen Uptake

Peak oxygen uptake ($\dot{V}$O_2peak_) was assessed during a progressive-intensity, treadmill (Trackmaster TMX425C, Newton, KS, United States) running exercise test on day 0 at SL using an indirect, open circuit respiratory system (True Max 2400, Parvo Medics, Sandy, UT, United States, calibrated to ambient pressure, temperature and humidity prevailing at each $\dot{V}$O_2peak_ test). The $\dot{V}$O_2peak_ test also was done once at HA (day 26, fourth full day at HA) to evaluate changes in $\dot{V}$O_2peak_ from SL. Participants fasted overnight (≥8 h) before testing. For each participant, the same metabolic cart was used for respiratory exchange measurements during both $\dot{V}$O_2peak_ tests.

### Steady-State Exercise Protocol

Substrate oxidation during steady-state exercise (treadmill walking) was assessed following the same protocol (**Figure 1B**) at SL, AHA, and CHA. Participants completed all three of the steady-state exercise tests following a 15-h fast, and all three exercise tests were performed at the same absolute $\dot{V}$O_2_. The absolute $\dot{V}$O_2_ (i.e., L/min) for the exercise experiments was selected to correspond to ∼55% of each individual’s absolute $\dot{V}$O_2peak_ at 4,300 m. Since the study schedule/design precluded measuring absolute $\dot{V}$O_2peak_ at 4,300 m before the participants completed the steady-state exercise test at SL, the absolute $\dot{V}$O_2peak_ at SL was used to establish the desired absolute $\dot{V}$O_2_ at which participants would exercise during the three steady- state exercise experiments. To calculate the desired absolute $\dot{V}$O_2_ for these tests, we assumed that absolute $\dot{V}$O_2peak_ at 4,300 m would be 27% lower than absolute $\dot{V}$O_2peak_ at SL (Young et al., 1985), so the target absolute $\dot{V}$O_2_ for the steady-state exercise experiments was calculated as follows: target absolute $\dot{V}$O_2_ = 0.55 × (0.73 × absolute $\dot{V}$O_2peak_ at SL). The absolute $\dot{V}$O_2peak_ measured on the fourth full day at HA was used to confirm the % of absolute $\dot{V}$O_2peak_ at which each participant exercised during the steady-state exercise experiments at AHA and CHA. The ACSM equations (American College of Sports Medicine, 2014) were used to predict the treadmill speed likely to elicit that target $\dot{V}$O_2_ while walking at a 2% grade. During the first week of phase 1 (SL days 1–6) and twice during phase 2 (HA days 7 and 14), participants completed familiarization trials using the same steady-state exercise protocol described below, except that blood and breath samples were not collected. Initially, they walked at a 2% grade and at the speed that had been predicted to elicit the target absolute $\dot{V}$O_2_. Respiratory exchange measurements during these walks were examined to determine whether the absolute $\dot{V}$O_2_ during walking was within ±5% of the target absolute $\dot{V}$O_2_, and if necessary, speed and/or grade adjustments were made during the first 20 min of exercise to ensure the desired absolute $\dot{V}$O_2_ was elicited.

Participants arrived at the laboratory at 05:00 h, voided, were weighed and had catheters placed in superficial forearm veins. They then sat semi-recumbent for ∼315 min while being infused with stable-isotope labeled amino acids for assessment of post-absorptive whole-body protein turnover in conjunction with other concurrent studies described elsewhere (Berryman et al., 2018). Approximately 20 min before the steady-state exercise experiment was scheduled to begin, the participants arose, voided their bladder (which started the timed urine collection for measurement of nitrogen excretion), and then stood quietly on the treadmill. Resting blood and breath samples were obtained 10 min before exercise, and then participants ingested 550 mL of the CHO or PLA drink. The participants then began walking for 80 min at a speed and grade selected to elicit the target $\dot{V}$O_2_. Participants consumed 300 mL of the carbohydrate or placebo drink after 20, 40, and 60 min of walking. Blood samples were obtained at 20, 40, 60, and 75 min of walking. Breath samples were collected every 10 min during the first 40 min of walking, and every 5 min during the last 40 min. Respiratory gas exchange (indirect calorimetry, True Max 2400, Parvo Medics, Sandy, UT, United States, calibrated to ambient pressure, temperature and humidity prevailing at each steady-state exercise experiment) was measured during the 5 min before the participants began walking, during the first 5 min of walking, and for 5 min at 20, 40, 60, and 75 min of walking. For each participant, the same metabolic cart was used for respiratory exchange measurements during all three of the steady-state exercise experiments. Rate of energy expenditure (kcal/min) was calculated from respiratory exchange measurements as described by Jeukendrup and Wallis (2005):

$$\begin{array}{c} Energy expenditure rate=(0.575\times {\dot{V}\mathrm{CO}}_{2})\;+\;(4.435\times {\dot{V}O}_{2}), \end{array}$$

where $\dot{V}$CO_2_ and $\dot{V}$O_2_ are in L/min. Immediately after completing the 80th min of steady-state exercise, participants voided their bladder (end timed urine collection period).

The carbohydrate drink consumed by CHO during the steady-state exercise trials contained corn-derived, crystalline fructose (KRYSTAR^®^ 300, Tate and Lyle Sugars, London, United Kingdom) and glucose (STALEYDEX^®^ 333, Tate and Lyle Sugars). During the 80 min steady-state exercise bouts, 1,450 mL total volume and 145 g total carbohydrate (62.25 g fructose + 79.75 g glucose for a 0.8 fructose-to-glucose ratio) were ingested, achieving an average carbohydrate ingestion rate of 1.8 g/min. We chose this beverage formulation so as to be consistent with current recommendations for the consumption of multiple transportable carbohydrates during endurance exercise (Jeukendrup, 2014). The natural ^13^C enrichment of the combined fructose-glucose drink was -11.03‰ ^13^C versus the internationally accepted standard for carbon isotope measures, Vienna Pee Dee Belemnite (VPDB), respectively. The carbohydrate drink was enriched with 200 mg U-^13^C-glucose (Cambridge Isotope Laboratories Inc., Tewksbury, MA, United States) to achieve a final enrichment of the glucose in the beverage of 213.5‰ δ ^13^C versus VPDB. This high ^13^C enrichment of the ingested glucose provides a strong signal in expired CO_2_, which negates the potential confounding effects of relatively small changes in background enrichment of CO_2_ observed from rest to exercise based on the experimental, Western-based diet (Gautier et al., 1993), and the relatively small contribution of the ingested naturally enriched fructose that is oxidized. Due to regulatory constraints on use of radioactive compounds in the Pikes Peak laboratory (located in a US National Park), we could not label one of the carbohydrates with ^13^C and the other with a radio isotope (i.e., ^14^C) to independently quantify glucose and fructose oxidation during the HA sojourn. Therefore, our measurements of ^13^CO_2_ in expired air only reflect the oxidation of the ingested glucose, and our data are reported as exogenous glucose oxidation rate.

### Measurement of Substrate Oxidation During Steady-State Exercise

Carbohydrate, fat, and protein oxidation rates were calculated from $\dot{V}$O_2_ (L/min), $\dot{V}$CO_2_ (L/min), and urinary nitrogen excretion rate (g/min) during the 80-min exercise bout as described by Jeukendrup and Wallis (2005):

$$\begin{array}{c} \mathrm{Carbohydrate}\;(g/min)=(4.344\times {\dot{V}\mathrm{CO}}_{2})-(3.061\times {\dot{V}O}_{2})-0.40\times \mathrm{urinary}\;\mathrm{nitrogen}; \end{array}$$

$$\begin{array}{c} \mathrm{Fat}\;(g/min)=(1.695\times {\dot{V}O}_{2})-(1.701\times {\dot{V}\mathrm{CO}}_{2})-1.77\times \mathrm{urinary}\;\mathrm{nitrogen}; \end{array}$$

$$\begin{array}{c} \mathrm{Protein}\;(g/min)=6.25\times [\mathrm{urine}\;\mathrm{nitrogen}\;\mathrm{excreted}\;(g)/\mathrm{urine}\;\mathrm{collection}\;\mathrm{duration}]. \end{array}$$

**FIGURE 2:**
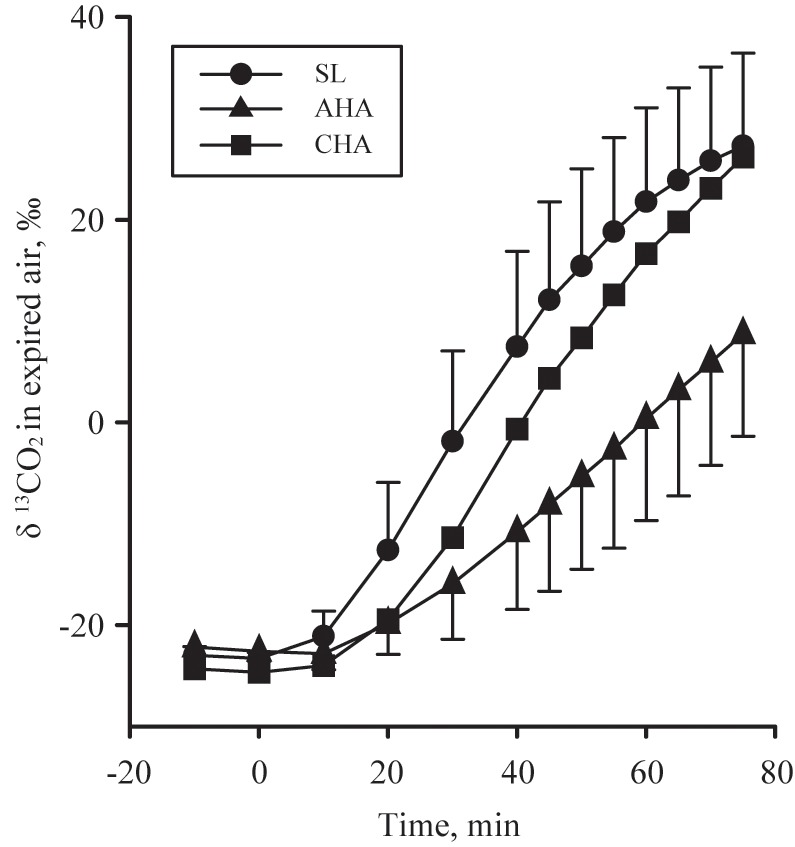
The expired δ ^13^CO_2_ versus VPDB in participants provided 1.8 g/min carbohydrate (CHO, *n* = 8, 65.25 g fructose + 79.75 g glucose, closed symbols, during 80-min steady-state exercise (treadmill walking) at sea level (SL) and at 4,300 m altitude, within 5–7 h after arrival (acute high-altitude, AHA) and again on the 22nd day of continuous residence at high-altitude (chronic high-altitude, CHA).

Calculation of protein oxidation assumed that nitrogen lost as sweat urea was negligible. The natural enrichment of the fructose-glucose ingested and the ^13^C/^12^C in expired gas samples were analyzed using isotope-ratio mass spectroscopy (Metabolic Solutions, Inc., Nashua, NH, United States). The isotopic enrichments were expressed in ‰ difference by comparison with VPDB (see **Figure 2** for expired δ ^13^CO_2_ versus VPDB during each endurance exercise substrate utilization protocol) and used to calculate exogenous glucose oxidation rate as follows:

$$\begin{array}{c} Exogenous glucose\mathrm{oxidation}(g/min)={\dot{V}\mathrm{CO}}_{2}[(R_{\exp}{-R}_{\mathrm{ref}})/(R_{\mathrm{exo}}{-R}_{\mathrm{ref}})]/k, \end{array}$$

where $\dot{V}$CO_2_ is in L/min, R_exp_ is the observed isotopic composition of expired CO_2_, R_ref_ is the isotopic composition of expired CO_2_ at rest before ingestion of the first dose of ^13^C-glucose, R_exo_ is the isotopic composition of the exogenous glucose ingested. The volume of CO_2_ provided by the complete oxidation of glucose is 0.747 L/g (k) (Trimmer et al., 2001; Ruzzin et al., 2003). Subtracting exogenous glucose oxidation from total carbohydrate oxidation yielded the amount of “other” carbohydrates oxidized. For PLA, the “other” carbohydrate oxidized was entirely comprised of endogenous carbohydrate (i.e., glucose provided by muscle and liver glycogen stores) oxidation. For CHO, the “other” carbohydrate oxidized was comprised of endogenous carbohydrate oxidation and exogenous fructose oxidation, the latter of which was not captured by the tracer techniques employed in the CHO group. These calculations assume that, in response to exercise, ^13^C labeled glucose is not irreversibly lost in pools of tricarboxylic acid cycle intermediates (Ruzzin et al., 2003) and/or bicarbonate (Trimmer et al., 2001) allowing for near complete ^13^CO_2_ recovery in expired gases. Due to the slow equilibration of the ^13^C/^12^C in expired CO_2_ (i.e., mouth) with ^13^C/^12^C in tissue derived CO_2_ (Pallikarakis et al., 1991), and to account for transient effects of hyperventilation (Jones and Jurkowski, 1979), only data and samples from the final 40 min of the steady-state exercise test were used to calculate expenditure and substrate oxidation (rates, g/min, and total amounts in 40 min, g).

### Blood and Urine Analyses

Serum glucose, free-fatty acids, glycerol, and plasma lactate concentrations were determined using enzymatic and colorimetric measurements (Beckman Coulter DXC 600 Pro, Beckman Coulter, Brea, CA, United States). Serum insulin concentrations were determined using an advanced automated immunoassay instrument (Immulite^®^ 2000: Siemens Healthcare Diagnostic, Deerfield, IL, United States). Glucose and insulin concentrations measured in the blood sample before the participants ingested the initial bolus of carbohydrate or placebo beverages and began exercising were used to calculate homeostasis model assessment of insulin resistance (HOMA-IR). Total urine volume produced during the timed collection was measured, and aliquots were frozen and stored at -20°C until analyzed. Nitrogen concentration of the urine was determined in triplicate using pyrochemiluminescence (Antek 9000, Houston, TX, United States), and nitrogen excretion was calculated by multiplying nitrogen concentration by volume of urine produced divided by collection duration.

### Statistical Analyses

As mentioned previously, this study was part of a larger investigation (Berryman et al., 2018) that was powered to detect body composition differences between standard and higher protein diets during concomitant altitude acclimatization and negative energy balance. However, eight participants per drink group (CHO vs. PLA) provided 80% power to detect differences in the rate of exogenous glucose oxidation with an expected mean difference of 0.25 g/min between treatment groups and between phases, assuming an alpha of 0.05 and a standard deviation of 0.17 g/min (Jeukendrup et al., 2006). We did not expect dietary protein level to affect substrate oxidation and blood measures at CHA (neither SL nor AHA were subject to the standard versus higher protein diet intervention). We confirmed that this was the case using linear mixed models with subject ID as a random effect, and dietary protein group, treatment (CHO, PLA), phase (SL, AHA, CHA), exercise time point, and their interactions as fixed effects. No significant effects of dietary protein group were found for any outcome except plasma lactate, for which a significant diet-by-treatment interaction was observed; however, the diet-by-treatment-by-phase interaction was not significant. Thus, the differences between dietary protein groups persisted across all phases, not just CHA, and as such are not a consequence of the dietary protein intervention, *per se*. Therefore, effects of the dietary protein intervention are not presented in this report, and the data were re-analyzed without dietary protein group in the model.

Linear mixed models were used to test the effects of treatment, phase, exercise time point, and their interactions on substrate oxidation and blood measures. Subject ID was treated as a random effect, and treatment, phase and time as fixed effects as appropriate per study outcome. These outcomes were modeled as doubly repeated measures with unstructured by unstructured or unstructured by autoregressive symmetry (based on lowest Bayesian information criterion) for phase and time point, respectively. Linear mixed models were also used to test the effects of treatment, phase and their interactions on cumulative substrate oxidation measures (i.e., no time effects). In this model, subject ID was treated as a random effect and treatment and phase as fixed effects. *Post hoc* analyses were adjusted for multiple comparisons using Bonferroni corrections. The alpha level for statistical significance is set at *P* ≤ 0.05. Data is reported as means ± SD unless otherwise specified. Statistical analyses were conducted using SAS 9.3 (SAS Institute Inc., Cary, NC, United States).

## Results

### Participant Characteristics

The participants were recreationally active (average $\dot{V}$O_2peak_ = 51.7 ± 7.4 mL/min/kg), non-obese (BMI = 26 ± 3; body mass = 82.8 ± 14.0 kg), young (age = 24 ± 6 years) men (**Table 1**). Dietary intakes during the sea level and high-altitude phases did not differ between groups (**Table 2**), with the exception of relative carbohydrate intake (CHO: 3.1 ± 0.3; PLA: 2.7 ± 0.2 g/kg/d, *P* < 0.05) and contribution of total energy derived from carbohydrate (CHO: 48 ± 1; PLA: 45 ± 1%, *P* < 0.05), which was attributable to ingestion of the carbohydrate beverage (145 g carbohydrate) during the CHA substrate oxidation experiment on day 22 at HA, and two additional practice trials (no substrate oxidation or blood measures) on days 7 and 14 at HA. Daily EIEE associated with individually prescribed exercise during the HA phase were the same for both groups, averaging 690 ± 178 kcal/d for the CHO and 695 ± 185 kcal/d for PLA.

**Table 1:** Detailed listing of physical characteristics of participants.

	CHO (*n* = 8)	PLA (*n* = 6)
**Characteristic**		
Age (years)	22.2 ± 2.0	26.6 ± 8.5
Height (cm)	178 ± 9	176 ± 6
Weight (kg)	82.3 ± 12.8	82.9 ± 16.6
BMI (kg/m^2^)	25.9 ± 3.0	26.7 ± 4.1
Body fat (%)	22.4 ± 5.4	25.0 ± 7.0
$\dot{V}$O_2peak_ (L/min) at SL	4.34 ± 0.74	4.06 ± 0.70
$\dot{V}$O_2peak_ (L/min) at HA	2.81 ± 0.38	2.75 ± 0.62

*Values are mean ± SD; peak O_*2*_ uptake ($\dot{V}$O_*2peak*_) at sea level (SL) and 4,300 m (HA). CHO participants ingested 1.8 g carbohydrate/min (65.25 g fructose + 79.75 g glucose) and PLA participants ingested placebo during steady-state exercise trials.*

**Table 2:** Detailed dietary intake for participants.

	SL, weight maintenance		HA, energy deficit	
	CHO	PLA	CHO	PLA
**Absolute intake (kcal/d or g/d)**			
Energy	2535 ± 485	2295 ± 333	2002 ± 230	1856 ± 231
Protein	92 ± 18	83 ± 18	134 ± 48	108 ± 31
Carbohydrate	339 ± 80	293 ± 62	238 ± 28	207 ± 26
Fat	92 ± 23	90 ± 17	62 ± 20	71 ± 20
**Relative intake (kcal/kg body mass/d or g/kg body mass/d)**			
Energy	31.5 ± 7.1	28.4 ± 6.5	26.1 ± 2.4	23.9 ± 2.3
Protein	1.2 ± 0.4	1.0 ± 0.1	1.7 ± 0.5	1.4 ± 0.5
Carbohydrate	4.2 ± 1.1	3.7 ± 0.3	3.1 ± 0.3	2.7 ± 0.2^∗^
Fat	1.1 ± 0.3	1.1 ± 0.2	0.8 ± 0.3	0.9 ± 0.2
**Percent energy intake (%)**			
Protein	15 ± 2	15 ± 4	27 ± 9	23 ± 7
Carbohydrate	53 ± 5	51 ± 7	48 ± 1	45 ± 1^∗^
Fat	32 ± 3	35 ± 4	28 ± 9	34 ± 8

*Values are mean ± SD for absolute (kcal/d, g/d), relative (kcal/kg body mass/d, g/kg body mass/d), and percent (%) energy intake; CHO participants (*n* = 8) ingested 1.8 g carbohydrate/min (65.25 g fructose + 79.75 g glucose) and PLA participants (*n* = 6) ingested placebo during steady-state exercise trials at sea level (SL) and high-altitude (HA). Participants in the CHO and PLA groups were further randomized to standard (1.0 g/kg/d) and higher (2.0 g/kg/d) protein diets beginning on day 2 at HA. Protein intake did not influence any study outcome; thus, dietary data are presented for CHO and PLA only. ^∗^Different (*P* < 0.05) from CHO during HA.*

Participants were weight stable during SL (day 1: 82.8 ± 14.0 kg and day 20: 83.3 ± 14.5 kg, ns), and lost (*P* < 0.05) 7.9 ± 1.9 kg of total body mass over the 22-day period of HA acclimatization and energy deficit. The participants exhibited an average 34% decrease in $\dot{V}$O_2peak_ at HA compared to SL. No differences were observed between treatments.

### Respiratory Gas Exchange During Steady-State Exercise

Resting peripheral blood oxygen saturation averaged 98.2 ± 1.3%, 85.3 ± 8.3%, and 89.5 ± 3.0% before exercise at SL, AHA, and CHA, respectively. There were no differences in $\dot{V}$O_2_, $\dot{V}$CO_2_ or energy expenditure measurements at the 40, 60, and 75 min of walking during any of the trials, indicating that the participants were in steady-state. There were no differences in $\dot{V}$O_2_ or energy expenditure (**Table 3**) between treatment groups or phases. Average $\dot{V}$O_2_ during the last 40 min of walking corresponded to 40% of $\dot{V}$O_2peak_ at SL and 60% of $\dot{V}$O_2peak_ at AHA and CHA. There were no differences in $\dot{V}$CO_2_ (**Table 3**) between CHO and PLA, but $\dot{V}$CO_2_ was lower at CHA than SL (*P* < 0.05).

**Table 3:** Ambient testing conditions during steady-state experiments, exercise intensity, respiratory gas exchange and energy expended during final 40 min of steady-state exercise.

	SL		AHA		CHA	
	CHO	PLA	CHO	PLA	CHO	PLA
Pressure^∗^ (mmHg)	761 ± 3	759 ± 2	459 ± 3	458 ± 2	459 ± 2	458 ± 2
Temperature (°C)	21.8 ± 0.9	21.8 ± 1.2	21.5 ± 2.3	19.8 ± 2.9	22.4 ± 2.0	21.8 ± 1.2
Humidity^∗^ (%)	50.3 ± 9.2	45.0 ± 7.8	34.4 ± 9.2	38.4 ± 5.0	32.9 ± 6.9	35.6 ± 5.1
Speed^∗^ (m/s)	1.8 ± 0.1	1.7 ± 0.2	1.6 ± 0.1	1.6 ± 0.2	1.7 ± 0.1	1.6 ± 0.2
Grade (%)	2.1 ± 0.4	1.8 ± 0.4	1.7 ± 0.8	1.8 ± 0.4	1.8 ± 0.5	1.9 ± 0.5
$\dot{V}$O_2_ (L/min)	1.77 ± 0.27	1.63 ± 0.28	1.68 ± 0.30	1.62 ± 0.25	1.71 ± 0.23	1.63 ± 0.27
$\dot{V}$CO_2_^∗^ (L/min)	1.64 ± 0.28	1.42 ± 0.29	1.53 ± 0.31	1.39 ± 0.26	1.47 ± 0.20	1.31 ± 0.22
RER^∗†^	0.93 ± 0.05	0.86 ± 0.06	0.91 ± 0.04	0.85 ± 0.04	0.86 ± 0.4	0.81 ± 0.08
Energy (kcal)	353 ± 51	322 ± 54	342 ± 62	324 ± 47	343 ± 46	318 ± 50

*Values are mean ± SD of ambient pressure, temperature, and humidity during experimental trials, walking speed, oxygen uptake ($\dot{V}$O_*2*_), carbon dioxide output ($\dot{V}$CO_*2*_), respiratory exchange ratio (RER), and total energy expenditure (energy) during final 40 min of 80 min steady-state exercise (treadmill walking) performed at sea level (SL, 55 m), at 4,300 m altitude ∼5 h after arrival (acute high-altitude, AHA), and on the 22nd day of continuous residence at high-altitude (chronic high-altitude, CHA) of participants who ingested 1.8 g carbohydrate/min (CHO, *n* = 8, 65.25 g fructose + 79.75 g glucose) or non-nutritive placebo (PLA, *n* = 6) during exercise. ^∗^Main effect (*P* < 0.05) of phase for pressure (SL > AHA and CHA), humidity (SL > AHA and CHA), walking speed (AHA < SL), $\dot{V}$CO_*2*_ (CHA < SL), and RER (CHA < SL). ^†^Main effect of treatment for RER (CHO > PLA).*

### Substrate Oxidation During Steady-State Exercise

**FIGURE 3:**
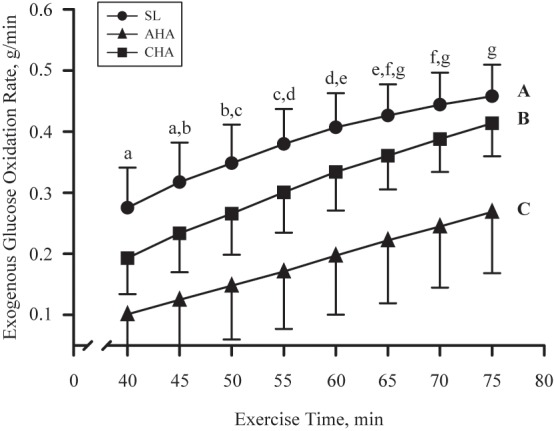
Mean ± SD of exogenous glucose oxidation rate (g/min) during last 40 min of 80-min steady-state exercise (treadmill walking) in participants provided 1.8 g/min carbohydrate (CHO, *n* = 8, 65.25 g fructose + 79.75 g glucose) at sea level (SL) and at 4,300 m altitude, within 5–7 h after arrival (acute high-altitude, AHA) and again on the 22nd day of continuous residence at high-altitude (chronic high-altitude, CHA). Points not sharing same letter are different (lower case = main effect of time, *P* < 0.05; upper case = main effect of phase, *P* < 0.05).

Total carbohydrate oxidation was greater (*P* < 0.05) in CHO than PLA for all three trials (**Table 4**). Total carbohydrate oxidation (g/40 min) was lower (*P* < 0.05) at CHA than at SL and AHA. For CHO, total exogenous glucose oxidation was less at AHA than at SL and CHA. The rate of exogenous glucose oxidation (g/min) in CHO (**Figure 3**) increased similarly over the final 40 min of each exercise trial across phases (main effect of time, *P* < 0.05). Exogenous glucose oxidation rate for CHO differed between all phases, being highest at SL, lowest at AHA and intermediate at CHA (main effect of phase, *P* < 0.05). Total other (as previously defined in Section “Materials and Methods”) carbohydrate oxidation (g/40 min, **Table 4**) was not different between CHO and PLA, but was lower (*P* < 0.05) at CHA than SL and AHA. Fat oxidation (g/40 min, **Table 4**) was greater (*P* < 0.05) in PLA than CHO during all three phases, and was greater at CHA than SL or AHA (*P* < 0.05). Protein oxidation was greater (*P* < 0.05) in CHO than PLA, but did not differ between SL, AHA, and CHA. No other treatment or phase effects were observed.

**Table 4:** Total substrate oxidized during final 40 min of steady-state exercise.

	SL		AHA		CHA	
	CHO	PLA	CHO	PLA	CHO	PLA
**Carbohydrate (g) glucose**						
Total^∗†^	68.3 ± 19.8	45.9 ± 21.4	59.7 ± 18.9	42.9 ± 15.7	45.5 ± 13.3	28.2 ± 17.9
Exogenous glucose^†^	15.3 ± 2.2	–	7.4 ± 3.7	–	12.4 ± 2.3	–
Other^†^	53.0 ± 20.1	45.9 ± 21.4	52.3 ± 16.9	42.9 ± 15.7	33.0 ± 12.6	28.2 ± 17.9
Fat^∗†^ (g)	7.5 ± 3.5	13.6 ± 6.6	9.1 ± 3.9	14.7 ± 3.5	15.2 ± 6.2	20.5 ± 8.5
Protein^∗^ (g)	3.1 ± 0.3	2.6 ± 0.8	3.1 ± 1.2	2.2 ± 0.3	3.4 ± 1.4	2.5 ± 0.4

*Values are mean ± SD of total (g) substrate oxidized during final 40 min of 80 min steady-state exercise (treadmill walking) performed at sea level (SL), at 4,300 m altitude ∼5 h after arrival (acute high-altitude, AHA), and on the 22nd day of continuous residence at high-altitude (chronic high-altitude, CHA) of participants who ingested 1.8 g carbohydrate/min (CHO, *n* = 8, 65.25 g fructose + 79.75 g glucose) or placebo (PLA, *n* = 6) during exercise. “Other” carbohydrate oxidation was calculated by subtracting exogenous glucose oxidation from total carbohydrate oxidation, and for PLA, was entirely comprised of endogenous carbohydrate (i.e., glucose provided by muscle and liver glycogen stores) oxidation, but for CHO, was comprised of endogenous carbohydrate oxidation and exogenous fructose oxidation, the latter of which was not captured by the tracer techniques employed in the CHO group. ^∗^Main effect (*P* < 0.05) of treatment on total carbohydrate (CHO > PLA), fat (CHO < PLA) and protein (CHO > PLA) oxidation; ^†^main effect (*P* < 0.05) of phase on total (CHA < SL, AHA) and other carbohydrate oxidation (CHA < SL, AHA), exogenous glucose oxidation (AHA < SL, CHA), and fat oxidation (CHA > SL, AHA).*

**FIGURE 4:**
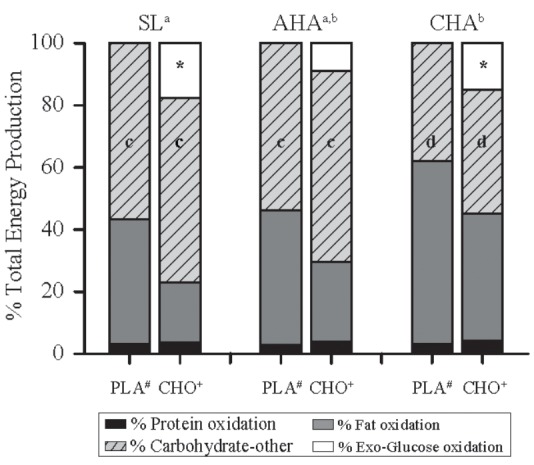
The percentage contribution from oxidation of different energy substrates to total energy production during the last 40 min of 80-min steady-state exercise (treadmill walking) in participants provided 1.8 g/min carbohydrate (CHO, *n* = 8, 65.25 g fructose + 79.75 g glucose) or non-nutritive placebo (PLA, *n* = 6) at sea level (SL) and at 4,300 m altitude, within 5–7 h after arrival (acute high-altitude, AHA) and again on the 22nd day of continuous residence at high-altitude (chronic high-altitude, CHA). For PLA, “other” carbohydrate oxidation is entirely endogenous carbohydrate (i.e., glucose provided by muscle and liver glycogen) oxidation, but for CHO, “other” carbohydrate is endogenous carbohydrate oxidation and exogenous fructose oxidation, the latter of which was not captured by the tracer techniques employed in the CHO group. ^a,b^Contribution from oxidation of fat was greater and from carbohydrate (exogenous glucose + other) less at CHA than at SL, but neither differed from AHA (main effect of phase, *P* < 0.05). ^∗^In CHO, contribution of exogenous carbohydrate oxidation was less at AHA than at SL or CHA, which did not differ (main effect of phase, *P* < 0.05). ^c,d^Contribution from oxidation other carbohydrates at SL and AHA did not differ, but was greater than at CHA. **^#^**Contribution of fat oxidation greater in PLA than CHO during all trials (main effect of treatment, *P* < 0.05). **^+^**Contribution of carbohydrate (exogenous glucose + other carbohydrate) oxidation was greater in CHO than PLA during all trials (main effect of treatment, *P* < 0.05).

The contribution of protein oxidation to total energy production during exercise did not differ between groups or phases and averaged 3.4 ± 0.9%, overall (**Figure 4**). Total carbohydrate oxidation accounted for a greater fraction of total energy production in CHO than PLA for all phases (*P* < 0.05), and carbohydrate oxidation contributed less to total energy production at CHA than SL (*P* < 0.05). In the CHO, exogenous glucose oxidation accounted for 18 ± 4% of total energy production at SL, but at AHA exogenous glucose oxidation accounted for much less (*P* < 0. 05), only 9 ± 4% of total energy production. At CHA, the contribution of exogenous glucose oxidation to total energy production in CHO was 15 ± 3%, which was greater than at AHA (*P* < 0.05) and not different from SL. The contribution from oxidation of other carbohydrates to total energy production did not differ between groups, but was less during exercise at CHA than for both SL and AHA (*P* < 0.05). Total fat oxidation accounted for a greater fraction of total energy production in PLA than CHO for all phases (*P* < 0.05), and fat oxidation contributed more to total energy production at CHA than SL (*P* < 0.05).

### Changes in Blood Metabolites During Steady-State Exercise

**FIGURE 5:**
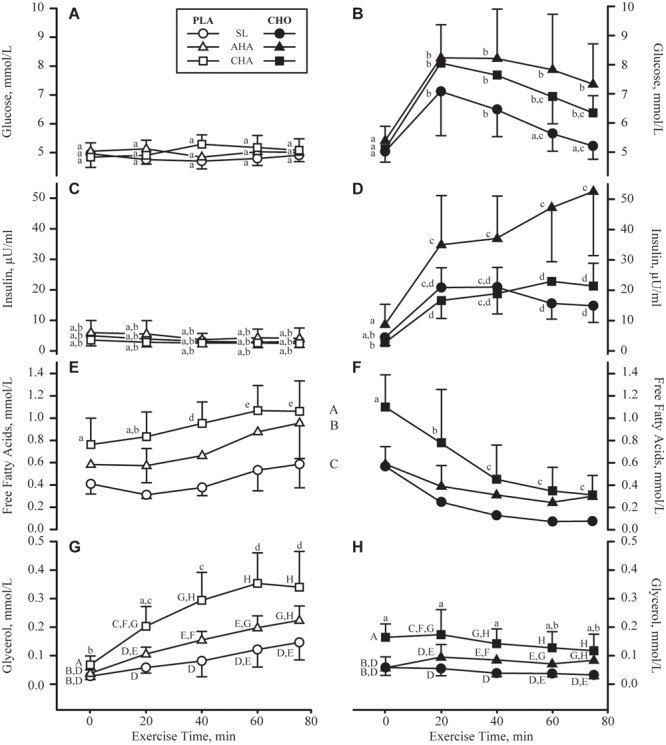
Mean + SD of serum concentrations of glucose (mmol/L, **A,B**), insulin (μU/mL, **C,D**), free fatty acids (mmol/L, **E,F**) and glycerol (mmol/L, **G,H**) concentrations in participants provided non-nutritive placebo (PLA, *n* = 6, open symbols, left column of panels) or 1.8 g/min carbohydrate (CHO, *n* = 8, 65.25 g fructose + 79.75 g glucose, closed symbols, right column of panels) during 80-min steady-state exercise (treadmill walking) at sea level (SL) and at 4,300 m altitude, within 5–7 h after arrival (acute high-altitude, AHA) and again on the 22nd day of continuous residence at high-altitude (chronic high-altitude, CHA). For glucose and insulin, points not sharing the same lower case letter are different (treatment by phase by time interaction, *P* < 0.05 for both metabolites). For free fatty acids, time points not sharing the same lower case letter are different (treatment by time interaction, *P* < 0.05), and phases not sharing same upper case letter are different (main effect of phase, *P* < 0.05), no interactions). For glycerol, time points not sharing the same lower case letter are different (treatment by time interaction, *P* < 0.05) and time points not sharing the same upper case letter are different (time by phase interaction, *P* < 0.05).

Serum glucose concentrations (**Figures 5A,B**) before exercise and beverage consumption did not differ between groups or phases. In PLA, glucose concentrations remained unchanged throughout the exercise bouts, and did not differ between phases. In contrast, glucose concentrations in CHO increased (*P* < 0.05) during the first 20 min of exercise in all three phases. By 60 min of exercise at SL, glucose concentrations in CHO had returned to pre-exercise levels, whereas glucose concentrations remained higher (*P* < 0.05) than pre-exercise levels throughout exercise at AHA and CHA. Serum insulin concentrations (**Figures 5C,D**) before exercise and beverage consumption were lower (*P* < 0.05) at CHA compared to AHA in CHO but not PLA. Insulin levels remained unchanged during exercise in PLA. The increase in insulin during exercise in CHO was more pronounced at AHA than at SL or CHA, with the differences achieving significance (*P* < 0.05) by 60 min. HOMA-IR did not differ between groups at any phase, and averaged (all participants) 1.04 ± 0.73 at SL, increasing (*P* < 0.05) to 1.82 ± 1.46 at AHA and then decreasing (*P* < 0.05) to 0.70 ± 0.34, which was not different from SL.

Serum free fatty acid concentrations (**Figures 5E,F**) before exercise and beverage consumption did not differ between groups or phases. However, during exercise, free fatty acid concentrations progressively increased (*P* < 0.05) in PLA, but progressively decreased (*P* < 0.05) in CHO. By 40 min of exercise and thereafter, free fatty acid concentrations were higher (*P* < 0.05) in PLA than CHO. In both CHO and PLA, free fatty acid concentrations were higher at AHA than SL, (*P* < 0.05) and higher at CHA than AHA (*P* < 0.05). Serum glycerol concentrations (**Figures 5G,H**) before exercise and beverage consumption were on average higher (main effect of treatment, *P* < 0.05) in CHO than PLA. Glycerol concentrations in PLA rose progressively through 60 min of exercise, and remained plateaued thereafter, but glycerol concentrations in CHO remained unchanged throughout exercise. In both groups, glycerol concentrations during exercise were highest at CHA, intermediate at AHA, and lowest at SL (*P* < 0.05).

**FIGURE 6:**
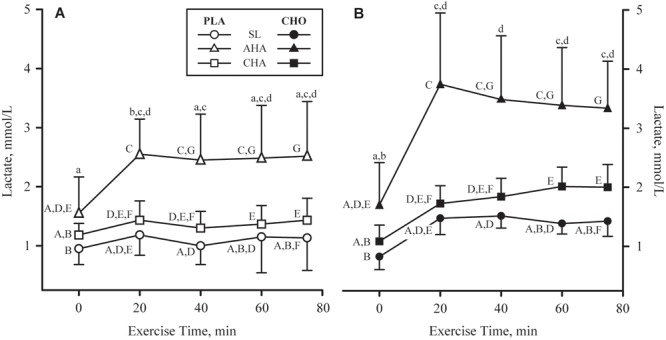
Mean + SD of plasma lactate concentrations in participants provided non-nutritive placebo (PLA, *n* = 6, open symbols, **(A)** or 1.8 g/min carbohydrate (CHO, *n* = 8, 65.25 g fructose + 79.75 g glucose, closed symbols, **(B)** during 80-min steady-state exercise (treadmill walking) at sea level (SL) and at 4,300 m altitude, within 5–7 h after arrival (acute high-altitude, AHA) and again on the 22nd day of continuous residence at high-altitude (chronic high-altitude, CHA). Time points not sharing the same lower case letter are different (treatment by time interaction, *P* < 0.05) and time points not sharing the same upper case letter are different (time by phase interaction, *P* < 0.05).

Plasma lactate concentrations (**Figures 6A,B**) before exercise and beverage consumption did not differ between groups, and were higher at AHA than SL (*P* < 0.05). Lactate concentrations increased in both groups during the first 20 min of exercise; in CHO, lactate concentrations remained higher than the resting value for the remainder of the exercise, but in PLA, lactate concentrations had declined after 40 min and were no longer different from resting values. In both groups, lactate concentrations rose higher during exercise at AHA than at SL or CHA. Lactate concentrations during exercise at CHA were higher than at SL after 60 min of exercise until the end of the bout.

## Discussion

This study investigated the effects of hypoxia, before and after development of altitude acclimatization, on oxidation of exogenous glucose ingested during steady-state exercise in healthy, young SL residents sojourning at HA while in negative energy balance. We demonstrated that exogenous glucose oxidation during metabolically matched exercise is suppressed by acute hypoxia compared to normoxic conditions, and that 21 days of HA acclimatization alleviates this hypoxic-suppression. Furthermore, the effects of acute and chronic hypoxia on blood glucose and insulin responses to exercise and carbohydrate ingestion that we observed raise the possibility that the concomitant changes in exogenous glucose oxidation may be related to adjustments in glycemic regulation.

In our CHO participants, 15.3 g of exogenous glucose were oxidized over the final 40 min of treadmill exercise at SL, but during exercise at same absolute metabolic rate at AHA 7.4 g of exogenous glucose was oxidized, over 52% less than at SL. Following 21 days of HA acclimatization, this hypoxic suppression of exogenous glucose oxidation was alleviated, and 12.4 g of exogenous glucose was oxidized, not different from that measured during metabolically matched exercise at SL. If we assume that fructose oxidation rate was 48% of the measured glucose oxidation rate (Jentjens et al., 2004; Rowlands et al., 2008), a reasonable estimate of exogenous carbohydrate oxidization in final 40 min of SL exercise would be 23 g. That is less than the exogenous carbohydrate oxidation rate measured by O’Hara et al. (2017) in participants exercising at SL and consuming a similar glucose and fructose mixture as the one our participants consumed. However, participants in that other study exercised at a substantially higher metabolic rate, 13 kcal/m, than our participants, who exercised at 9 kcal/min. The higher exercise intensity (Pirnay et al., 1982) used in that investigation would account, at least in part, for the higher exogenous carbohydrate oxidation rates compared to our study. Regardless of those differences, the finding that the hypoxic suppression of exogenous glucose oxidation was alleviated following 21 days of HA acclimatization, and exogenous glucose oxidation rates had returned to levels not different from those measured during similar exercise at SL, has to our knowledge not been previously reported.

It was surprising that total amount of carbohydrate oxidized was the same at AHA as SL in both groups, given that several previous studies of altitude effects on substrate utilization during exercise (Brooks et al., 1991; Roberts et al., 1996; Lundby and Van Hall, 2002; O’Hara et al., 2017) have reported that carbohydrate utilization increases during exercise at HA compared to exercise at SL. We speculate that the differences between our observations and previous reports may not only reflect differences in exercise intensity, but also differences in the exercise mode. Previous studies that observed increased carbohydrate utilization at HA compared to sea level employed cycling exercise, whereas in our study, participants walked on a treadmill. Even in the studies in which cycling exercise was performed at very similar rates of whole-body energy expenditure as in ours (Brooks et al., 1991; Roberts et al., 1996; Lundby and Van Hall, 2002), that energy expenditure is distributed over a smaller mass of muscle during cycling exercise than during walking. The higher metabolic rate maintained by the smaller volume of actively contracting muscle fibers during cycling exercise as compared to walking is likely to elicit higher rates of carbohydrate oxidation during exercise, and that effect is likely to be even greater at HA when the relative intensity of exercise ($\dot{V}$O_2max_) is increased compared to at SL.

Acute hypoxia alters glycemic regulation, in particular insulin sensitivity is decreased (Larsen et al., 1997; Braun et al., 2001; Oltmanns et al., 2004; Peltonen et al., 2012), whereas chronic hypoxia (altitude acclimatization) at least partially alleviates that effect (Brooks et al., 1991; Larsen et al., 1997; Braun et al., 1998), and those changes in insulin sensitivity modulate changes in blood glucose homeostasis. The changes in HOMA-IR exhibited by both PLA and CHO during altitude sojourn, and the blood glucose and insulin responses observed in CHO and PLA groups during hypoxic exercise before and after HA acclimatization are consistent with those reported effects of hypoxia on glycemic regulation.

Ingestion of exogenous carbohydrate is well documented to increase the contribution of carbohydrate oxidation and decrease the contribution of fat oxidation to total energy production during exercise at SL (Jentjens et al., 2004), and our observations during exercise at HA are consistent with that. However, since fat oxidation contributed more to total energy production during exercise at AHA in PLA than CHA, the question might arise as to why the suppression of exogenous glucose oxidation in CHO during exercise at AHA was not offset by an increased contribution of fat oxidation. During SL exercise, hyperglycemia and hyperinsulinemia have been shown to inhibit lipolysis and mobilization of fatty acids, as well as oxidation of intramuscular fatty acids and triglycerides (Coyle et al., 1997). Our observations suggest a similar effect at HA, in that circulating free fatty acids concentrations in CHO declined during exercise both at SL and at HA, but increased in PLA. Thus, during exercise under conditions of acute hypoxia, ingesting exogenous carbohydrate provides less of an advantage in terms of sparing endogenous glucose stores from oxidation, since exogenous glucose oxidation is suppressed. Further, the resulting high levels of blood glucose and insulin likely inhibit fat oxidation, forcing energy requirements to be achieved with greater reliance on endogenous carbohydrate oxidation. During exercise at CHA, not only was the suppression of exogenous glucose oxidation alleviated, but the lower blood glucose and insulin concentrations during exercise appears to have enabled a greater contribution of fat oxidation to total energy production in CHO as well as PLA, compared to SL and AHA.

Certain aspects of our experimental approach should be considered to appropriately interpret our findings in the context of previous research. Our sample size, albeit sufficient to test our hypothesis, can be considered small, which may limit the extension of our findings. Further, our participants maintained negative energy balance and lost ∼8 kg body mass over the 21-day HA acclimatization phase. Since the SL and AHA experiments had already been completed before negative energy balance began, those experiments were not affected by the energy deficit. Including fully fed, energy balanced control groups in the design was logistically unfeasible and outside the scope of our study (i.e., test effects of AHA exposure and energy deficit during HA acclimatization), so we cannot rule out the possibility that body mass and fat loss affected the metabolic responses observed during the CHA exercise tests. Thus, our conclusion that altitude acclimatization alleviates the blunting of exogenous glucose oxidation produced by acute hypoxia requires confirmation in fully fed participants maintaining stable body weight during HA acclimatization. Another consideration is that we enriched the carbohydrate beverage with ^13^C-glucose, but did not enrich the fructose component. Therefore, we could not measure total exogenous carbohydrate oxidation, but only exogenous glucose oxidation.

In summary, our study demonstrated that exogenous glucose oxidation during exercise is suppressed by acute hypoxia, but after 21 days of altitude acclimatization and energy deficit, that suppression is alleviated. Whether altitude acclimatization alleviates hypoxic suppression of exogenous glucose oxidation in fully fed sojourners maintaining stable body weight remains to be demonstrated. From a practical perspective, our observations suggest that traditional recommendations for consuming carbohydrate during exercise may not have the same effects on substrate oxidation during exercise on the first few days of a HA sojourn as during exercise at SL, but following a period of HA acclimatization, metabolic effects of consuming exogenous carbohydrate during exercise may normalize.

## Author Contributions

AY, CB, and SP conceived the study question and designed the experiments. AY, CB, RK, AD, LM, MW, CC, NM, JC, JR, and SP performed the experiments. CB, AD, and CC analyzed the data. AY, CB, RK, and SP interpreted the data. AY, CB, and SP prepared the figures and drafted the manuscript. All authors edited and revised the manuscript and approved the final version.

## Conflict of Interest Statement

The opinions or assertions contained herein are the private views of the authors and are not to be construed as official or as reflecting the views of the Army or the Department of Defense. Any citations of commercial organizations and trade names in this report do not constitute an official Department of the Army endorsement of approval of the products or services of these organizations. The authors declare that the research was conducted in the absence of any commercial or financial relationships that could be construed as a potential conflict of interest.
